# Nucleotide excision repair (NER) alterations as evolving biomarkers and therapeutic targets in epithelial cancers

**DOI:** 10.18632/oncoscience.283

**Published:** 2015-12-29

**Authors:** Kent W. Mouw, Alan D. D’Andrea, Panagiotis A. Konstantinopoulos

**Affiliations:** Dana-Farber Cancer Institute, Harvard Medical School, Boston, MA 02215, USA

**Keywords:** Nucleotide excision repair (NER), ovarian cancer, bladder cancer, DNA repair, platinum

The nucleotide excision repair (NER) pathway is a highly conserved and remarkably versatile DNA repair pathway that functions to identify and repair bulky intrastrand DNA crosslinks generated by a variety of genotoxic agents including platinum drugs. Platinum analogs are active anticancer agents and constitute the backbone of first-line chemotherapy used in a number of epithelial malignancies. Given the role of the NER pathway in repair of platinum-induced DNA damage, a number of studies have recently investigated the prevalence and of NER pathway alterations in various epithelial tumors as well as their association with response to platinum and other chemotherapy agents.

In epithelial ovarian cancer (EOC), analysis of the TCGA data set revealed that nearly half of all EOCs harbor an alteration in the homologous recombination (HR) pathway, which includes *BRCA1* and *BRCA2* (*BRCA1/2*). In addition, 8% of high-grade serous EOCs have an alteration (non-synonymous mutation, splice site mutation, promoter hypermethylation, or homozygous deletion) of at least one gene in the NER pathway, and half of these NER alterations (4% of tumors overall) occurred in the absence of a known HR alteration. [1] Patients with tumors harboring NER alterations had improved overall and progression-free survival compared to tumors without NER or *BRCA1/2* alterations. Given that nearly all EOC patients receive platinum-based chemotherapy, and because the durability of platinum response is closely associated with survival in these patients, the improved survival of patients with NER-altered tumors likely reflects increased platinum sensitivity in this population. Moreover, survival of patients with NER-altered tumors was similar to patients with tumors harboring *BRCA1/2* alterations, suggesting that NER pathway alterations may contribute to EOC platinum sensitivity to an extent similar to the effect of *BRCA1/2* loss.

Importantly, unlike *BRCA1/2* alterations, NER pathway alterations were not associated with sensitivity to poly(ADP-ribose) polymerase (PARP)-inhibitors. Functional analysis of several NER mutations identified in the TCGA cohort revealed that expression of the mutant in a NER-deficient background failed to rescue cisplatin sensitivity, whereas no difference in PARP-inhibitor sensitivity or HR activity was noted. Together, these findings identify an important subset of NER-deficient, HR-proficient EOCs with discordant platinum and PARP-inhibitor profiles, and underscore the potential role of NER pathway alterations as predictive biomarkers of response to specific anticancer therapies.

**Figure 1:**
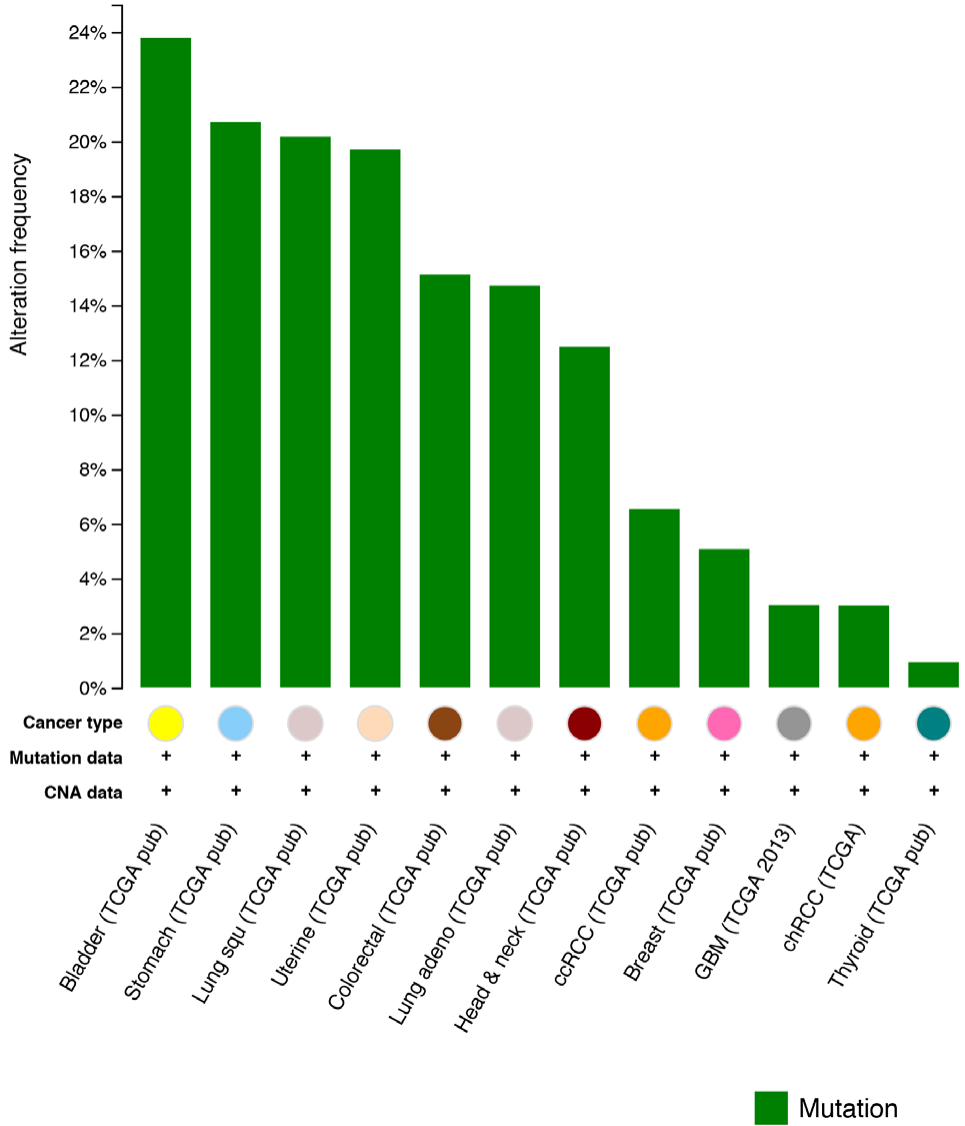
Frequency of NER pathway gene mutations in several solid tumor types Figure generated using the cBioPortal (http://www.cbioportal.org).[4]

More broadly, NER pathway alterations are also being identified in other epithelial tumor types and may serve as important biomarkers in diverse clinical settings where platinum agents are commonly employed. In urothelial carcinoma, we and others recently identified recurrent *ERCC2* mutations, and show that *ERCC2*- deficient tumors have increased response rates to cisplatin- based chemotherapy regimens. [2, 3] Review of the TCGA dataset using the cBIO portal reveals presence of NER alterations in several other epithelial cancers [4] (Figure 1), and additional studies may identify other clinical contexts in which NER pathway alterations could be used to inform therapy selection.

Finally, it is important to note that NER pathway alterations may also render tumors susceptible to novel anticancer therapies such as targeted immunotherapies and cell cycle checkpoint inhibitors. Given that NER- mutated tumors harbor higher mutational loads, they may possess more tumor-specific neoantigens, resulting in increased number of tumor-infiltrating lymphocytes (TILs) [5]. Several studies have shown that hypermutated tumors such as mismatch repair (MMR)-deficient or POLE-mutated tumors are associated with higher neoantigen loads and an elevated number of TILs, which is counterbalanced by overexpression of immune checkpoints such as PD-1/PD-L1 AND CTLA4 [6]. Such hypermutated tumors have been shown to particularly susceptible to immune checkpoint inhibitors targeting the PD-1/PD-L1 or CTLA4 pathways [7]. Finally, NER-deficient tumors are particularly sensitive to cell cycle checkpoint inhibition, in particular inhibitors of the ATR-CHK1-WEE1 pathway. ATR is activated by DNA single-strand–double-strand junctions that arise as intermediates in NER and triggers the intra-S phase and the G2 checkpoints via phosphorylation of CHK1 [8]. Activated Chk1 in turns phosphorylates WEE1 (resulting in its activation) and cell division cycle 25 (CDC25A and CDC25C) phosphatases (resulting in their inhibition) to inhibit cell cycle progression through the coordinated suppression of cyclin-dependent kinase activity. Several approaches to inhibit the ATR/CHK1/WEE1 pathway including ATR inhibitors (such as VX-970 and AZD6738), WEE1 inhibitors (such as AZD1775) and CHK1 inhibitors (GDC-0425 and LY2606368) are currently in early clinical trial evaluation in a number of epithelial cancers, and NER alterations may serve as a useful biomarker to define a subset of tumors that are particularly sensitive to these agents.
